# Comparative Analysis of the Nutrient Composition of *Caulerpa lentillifera* from Various Cultivation Sites

**DOI:** 10.3390/foods14030474

**Published:** 2025-02-01

**Authors:** Wenchuan Zhou, Yun Wang, Rui Xu, Jialin Tian, Ting Li, Suwen Chen

**Affiliations:** 1 Shenzhen Fisheries Development Research Center, Shenzhen 518067, China; zhouwenchuan163@163.com; 2 Key Laboratory of Aquatic Product Processing, Ministry of Agriculture and Rural Affairs, South China Sea Fisheries Research Institute, Chinese Academy of Fishery Sciences, Guangzhou 510300, China; xr497252775@163.com (R.X.); jltian2023@163.com (J.T.); liting@scsfri.ac.cn (T.L.); chensuwen407@163.com (S.C.); 3 Key Laboratory Efficient Utilization and Processing of Marine Fishery Resources of Hainan Province, Hainan Engineering Research Center of Deep-Sea Aquaculture and Processing, Sanya Tropical Fisheries Research Institute, Sanya 572018, China

**Keywords:** *Caulerpa lentillifera*, nutrient composition, amino acid, fatty acid

## Abstract

The nutrient, amino acid, and fatty acid compositions of *Caulerpa lentillifera* from various aquaculture regions were assessed to analyze their nutritional characteristics and potential for aquaculture development. The nutrient composition of *C. lentillifera* was determined according to the standard national nutrient determination methods of the Association of Official Analytical Chemists, and the following data were revealed. (1) The basic nutritional components of *C. lentillifera* were relatively more abundant in the three aquaculture areas in Guangdong Province. The crude protein content in *C. lentillifera* was measured at (8.70 ± 0.36)% and (18.57 ± 1.59)% for samples collected from the Dapeng and Daya areas, respectively. These values were significantly higher compared to those obtained from the Beihai, Dongshan, and Nha Trang regions (*p* < 0.05). Additionally, the crude lipid content of *C. lentillifera* from the Daya population was measured at (5.10 ± 1.59)%, which was significantly higher than that of samples from the Beihai, Dongshan, and Nha Trang areas (*p* < 0.05). Furthermore, the total sugar contents in *C. lentillifera* were quantified as (32.50 ± 4.22)% and (32.87 ± 2.59)% for samples from the Daya and Shanwei regions, respectively. These values were significantly greater (*p* < 0.05) compared to those observed in the other four populations. (2) The total amino acid (TAA) content of *C. lentillifera* ranged from 7.05% to 12.37%, with notable concentrations of the fresh amino acids aspartic acid (Asp) and glutamic acid (Glu). Significant variations in the TAA and essential amino acid (EAA) levels were observed among the cultivation regions (*p* < 0.05), with the Shanwei population exhibiting the highest TAA content of 12.37% and EAA content of 4.65%, surpassing all other populations except for Guangdong Province (*p* < 0.05). (3) The fatty acid composition analysis revealed that the total fatty acid (TFA) and unsaturated fatty acid (UFA) levels in the long-stemmed grape fern alga from Daya were 2.400% and 1.048%, respectively, and significantly greater than those in the other populations except for Dapeng (*p* < 0.05). These results imply that the nutritional quality of the Daya population of *C. lentillifera* is relatively high. *C. lentillifera* exhibits a palatable flavor profile, making it suitable for consumption and the development into high-quality seafood condiments, thereby contributing to the environmentally sustainable advancement of *C. lentillifera* aquaculture.

## 1. Introduction

*Caulerpa lentillifera*, commonly known as sea grape, is a type of green macroalgae that falls under the categories of Chlorophyta, Ulvophyceae, Bryoposodales, Caulerpaceae, and *Caulerpa*. *C. lentillifera* can be found throughout Southeast Asia, Okinawa Japan, Thailand, Oceania, and South Korea, as well as in tropical and subtropical marine environments [1]. As reported in the literature, this green seaweed was initially recorded on the Red Sea coast and later found in numerous other places, particularly in the Indo-Pacific region [2]. Its body is bright green, and due to its solid apparatus, stolons, and upright branches, which are distributed on symmetrical cysts, its shape resembles a bunch of grapes; thus, *C. lentillifera* is also known as the sea grape [3]. In several Asian countries, *C. lentillifera* is often made into a salad because of its tasty flavor. Moreover, *C. lentillifera* is rich in amino acids, unsaturated fatty acids (UFAs), vitamins, minerals, and a variety of polysaccharides and it has the advantages of low liquid and low calorie contents but high protein and dietary-fiber contents. Additionally, *C. lentillifera* has a rich and juicy taste, similar to caviar; thus, it is also known as green caviar [4,5]. Furthermore, *C. lentillifera* contains a variety of active substances. Studies have shown that its extract has strong antioxidant activity and free radical scavenging capacities; immunomodulatory and antitumor activities; anticoagulant effects; and blood sugar and lipid regulatory properties [6,7,8]. The researchers identified the monosaccharide components and structural characteristics of the polysaccharides in *C. lentillifera* using infrared spectroscopy, high performance liquid chromatography (HPLC), and nuclear magnetic resonance (NMR) analyses. Their findings indicated that the polysaccharides predominantly comprised mannose, galactose, and xylose, with some also containing glucose and glucosamine [9]. The critical active compound, caulerpin, is a red bisindole alkaloid frequently found in various marine green algae, including *Caulerpa racemose*, *Caulerpa prolifera*, and *Caulerpa sertularioides* [10]. Due to its rich nutrient profile and bioactive compound composition, the demand for *C. lentillifera* has recently increased in several Asian countries, leading to a rise in market prices. However, the present production rate of *C. lentillifera* is insufficient to satisfy the existing demand. The suboptimal cultivation conditions for the alga may contribute to its reduced productivity. Consequently, it is imperative to determine the optimal culture conditions to enhance the productivity of *C. lentillifera*.

In recent years, artificial breeding has been introduced in Shandong, Fujian, Guangdong, Hainan, Taiwan, and other regions via a variety of breeding modes, including sea culture, land-based culture, and pond culture [11,12]. The growth of *C. lentillifera* has been a major factor in the development of this species. To investigate the quality of *C. lentillifera* cultured in different areas, the nutrient composition (moisture, crude protein, crude liquid, ash, mannitol, and iodine), amino acid composition, fatty acid composition, and mass fractions of *C. lentillifera* from six culture groups in four regions (Fujian, Guangdong, Guangxi, and Vietnam) were determined, and the nutrient compositions were compared to better understand the nutrients in *C. lentillifera*. This study aims to conduct a comprehensive evaluation of the nutritional value of *C. lentillifera* across various regions, thereby contributing to the advancement of environmentally sustainable cultivation and utilization practices for this species.

## 2. Materials and Methods

### 2.1. Sample Collection

*C. lentillifera* samples were collected from six farms in Guangdong, Fujian, Guangxi, and Vietnam. The sampling locations are shown in Table 1. The samples were collected between 17 June 2022 and 27 October 2022. They were transported to the laboratory in a viable state, thoroughly rinsed with seawater to remove surface impurities, and prepared for experimental use. Subsequently, the samples were evenly divided into three portions, weighed, and stored in a freezer at −80 °C.

### 2.2. Methods by Which the Basic Nutrient Composition Was Determined

The basic nutrient composition of *C. lentillifera* was determined according to the standard methods of the Association of Official Analytical Chemists [13]. For moisture determination, samples were dried at 105 °C for 72 h to determine the moisture. The dried samples were pulverized into a fine powder. Two grams of the powder were utilized for ash measurement in a muffle furnace (FO610C, Yamato Scientific Co., Ltd., Tokyo, Japan) at 550 °C, 1 g was employed for the determination of crude lipids using the Soxhlet extraction method (Soxtec 2055, FOSS, Höganäs, Sweden), and 0.2 g were used for the assessment of crude protein via the Kjeldahl method (Kjeltec 8400, FOSS, Hoganos, Sweden). Additionally, the following methods were used to evaluate the other nutrients: total sugar content (GB/T 15672-2009) [14], crude fiber content (GB/T 5009.10-2003) [15], iodine content (GB 5009.267-2016) [16], mannitol content (SC/T 3405-2018) [17], total amino acid (TAA) content (GB 5009.124-2016) [18], and fatty acid content (GB 5009.168-2016) [19].

The TAA were quantified using the method of GB 5009.124-2016. The appropriate amount of mixed samples (100 mg) was accurately weighed, and 5 mL of a 1:1 hydrochloric acid solution was added to the hydrolysis tube. It was mixed thoroughly, and the tube was placed in an electric blast thermostat set at 110 ± 1 °C for hydrolysis over a period of 22 h. After hydrolysis, the tube was removed and allowed to cool to room temperature. The hydrolysis tube was opened, and the hydrolysate was filtered into a 10 mL volumetric flask. The hydrolysis tube was rinsed several times with a small amount of water, transferring the rinsing solution into the same volumetric flask. The volume was adjusted to the calibration mark with water and mixed thoroughly. A pipette of 0.05 mL of the filtrate was accurately measured into a 15 mL test tube and was evaporated to dryness under a nitrogen stream. The residue was reconstituted in 2 mL of 0.02 mol L^−1^ hydrochloric acid solution, with thorough mixing being ensured. The solution was passed through a 0.22 μm microporous filter membrane before proceeding with the analysis. The quantification of TAA was conducted utilizing an automated amino acid analyzer (LA8080, Hitachi, Tokyo, Japan).

The fatty acid profile was performed using the method of GB 5009.168-2016. For fat extraction, an appropriate quantity of the sample was weighed into a 100 mL cuvette, followed by the addition of 8 mL of water. The mixture was thoroughly homogenized before adding 10 mL of hydrochloric acid, ensuring thorough mixing. The flask was then placed in a water bath maintained at 80 °C for hydrolysis, which was conducted for 1 h. The flask was agitated every 30 min to ensure that particles adhering to the flask walls were incorporated into the solution. Upon the completion of hydrolysis, the sample was removed and allowed to cool to room temperature. Subsequently, 10 mL of 95% ethanol was added to the sample and mixed thoroughly. Fat extraction was performed using 100 mL of ether in three separate batches, with the extracts being combined into a 100 mL flat-bottomed flask. The ether layer was evaporated to yield the fat. For the saponification of fat and the methyl esterification of fatty acids, to the fat extract, 4 mL of a 2% sodium hydroxide methanol solution was added, and the mixture was incubated in a water bath at 45 °C for 30 min. Subsequently, 4 mL of a 14% boron trifluoride methanol solution was added, and the mixture was again incubated in a water bath at 45 °C for 30 min. After the water bath treatment, the mixture was allowed to cool to room temperature. In a centrifuge tube, 3 mL of n-hexane was added, and the mixture was subjected to extraction by shaking for 2 min, followed by a period of settling to allow for phase separation. The supernatant was collected and filtered through a 0.45 μm membrane for analysis. The samples were analyzed using an Agilent 7890A gas chromatograph (Aglient Technologies, Palo Alto, CA, USA).

### 2.3. Analysis of the Amino Acid Composition

The amino acid score (AAS) was calculated via the standard essential amino acid (EAA) model score proposed by the FAO/WHO [20], and the amino acid model of egg protein proposed by the Institute of Nutrition and Food Safety of the Chinese Academy of Preventive Medical Sciences (IFSS) was used to calculate the chemical score (CS) [21] and essential amino acid index (EAAI).

### 2.4. Data Analysis

There were three parallel treatment groups at each sampling point. The data are expressed as the means ± standard deviations ($\bar{x}$ ± SDs). All the data were subjected to one-way analysis of variance (ANOVA) in SPSS 26.0 to determine significance, and differences between groups were analyzed by multiple comparisons via Duncan’s method, and *p* < 0.05 was considered to indicate a significant difference.

## 3. Results

### 3.1. Comparison of the C. lentillifera Basic Nutrient Compositions

The basic nutrient compositions of *C. lentillifera* from different regions are shown in Table 2. The *C. lentillifera* moisture contents were extremely high, close to 95%, and there was no significant difference between the populations (*p* > 0.05). The ash determination results revealed that the Nha Trang population had the highest ash content (65.50%), the Shanwei population had the lowest content (22.83%), and the ash contents of the Beihai, Dongshan, and Nha Trang *C. lentillifera* populations were significantly greater than those of the three Guangdong populations (*p* < 0.05). In terms of crude protein content, the Dapeng population had the highest (18.70%), the Nha Trang population had the lowest (12.53%), and the populations followed the order Dapeng Bay > Daya Bay > Shanwei > Dongshan > Beihai > Nha Trang; additionally, the crude protein contents of the *C. lentillifera* samples from Dapeng Bay and Daya Bay were significantly greater than the samples from the Beihai, Dongshan, and Nha Trang populations (*p* < 0.05). It was also revealed that the Daya Bay population had the highest crude liquid content (5.10%), and the Shanwei population had the lowest content (3.83%). The crude liquid content of the Daya Bay population was significantly greater than that of the Beihai, Dongshan, and Nha Trang populations (*p* < 0.05). Moreover, the Shanwei population presented the highest total sugar content (32.87%), the Nha Trang population presented the lowest content (18.13%), and the Daya Bay and Shanwei populations presented relatively high total sugar contents, which were significantly greater than those of the other four populations (*p* < 0.05). The mannitol determination results revealed that the DongShan population presented the highest mannitol content (7.47%), the Dapeng Bay population presented the lowest content (5.30%), and the Shanwei and DongShan populations presented significantly greater mannitol contents than the Dapeng Bay population (*p* < 0.05). Finally, the iodine content of the Nha Trang *C. lentillifera* population was significantly greater than that of the other populations (*p* < 0.05). Among the six *C. lentillifera* populations, the Guangdong population presented a greater content of basic nutrients and was considered to be better quality than the other three regional populations.

### 3.2. Analysis of the Amino Acid Composition of C. lentillifera

The amino acid compositions and mass fractions of the different populations of *C. lentillifera* are shown in Table 3. A total of 17 amino acids were detected in the *C. lentillifera* samples; notably, tryptophan was not detected. The TAA content of *C. lentillifera* ranged from 7.05% to 12.37%, the EAA content ranged from 2.89% to 4.65%, the nonessential amino acid (NEAA) content ranged from 4.15% to 7.87%, and the flavor amino acid (DAA) content ranged from 3.52 to 5.31%. The most abundant NEAA was aspartic acid (Asp, ranging from 0.80 to 1.32%), followed by glutamic acid (Glu, ranging from 0.85 to 1.21%). There were no significant differences (*p* > 0.05) among the TAA, EAA, and NEAA contents of the three populations of long-stemmed grape ferns in Guangdong, although the TAA and DAA contents in the Daya Bay and Shanwei populations were significantly greater than those in the Beihai, Dongshan, and Nha Trang populations (*p* < 0.05). Additionally, the EAA contents in the Dapeng Bay and Shanwei samples were significantly greater than those in the Beihai, Dongshan, and Nha Trang samples (*p* < 0.05), while the NEAA contents in the Dapeng Bay, Daya Bay, and Shanwei samples were significantly greater than those in the Beihai, Dongshan, and Nha Trang samples (*p* < 0.05). Furthermore, the EAA/TAA ratios of the six populations of long-stemmed grape ferns ranged from 35.87% to 41.03%, the EAA/NEAA ratio ranged from 55.93 to 69.63%, and the DAA/TAA ratio ranged from 41.00–50.13%. The EAA/TAA, EAA/NEAA, and DAA/TAA ratios of *C. lentillifera* from Beihai, Dongshan, and Nha Trang were approximately 40%, 70%, and 50%, respectively, with no significant differences among these three regions (*p* > 0.05), but all were significantly greater than the corresponding ratios from the Guangdong population (*p* < 0.05).

### 3.3. A comprehensive Assessment of the Amino Acid Profile in C. lentillifera

The nutritional quality of the *C. lentillifera* cultivated in six different areas was evaluated according to the EAA evaluation criteria proposed by the FAO/WHO, and the results are shown in Table 4. The AASs revealed that the first *C. lentillifera*-limiting amino acid was methionine (Met) + cystine (Cys), and the second limiting amino acid was isoleucine (Ile); moreover, the amino acid with the highest score among the six different samples was phenylalanine (Phe) + tyrosine (Tyr). Additionally, the chemical score data revealed that the first limiting amino acid of *C. lentillifera* from all populations was Met + Cys, while the second limiting amino acid varied among the six regions. The second limiting amino acid of the Daya Bay and Beihai populations was Ile, which was in agreement with the AASs, but the second limiting amino acid of the other four populations was lysine (Lys). The amino acid with the highest chemical score differed among the six regions, as threonine (Thr) gave the highest chemical score in the Guangdong region while Phe + Tyr gave the highest scores in the other three region, which is consistent with the AASs. The highest EAAI was found in the Nha Trang, Vietnam, population among the six regions, while the lowest EAAI was found in the Dongshan, Fujian population.

### 3.4. Analysis of the Fatty Acid Composition of C. lentillifera

The fatty acid compositions and mass fractions of different populations of *C. lentillifera* are shown in Table 5. A total of 13 fatty acids were detected in *C. lentillifera*, including four saturated fatty acids (SFAs), three monounsaturated fatty acids (MUFAs), and six polyunsaturated fatty acids (PUFAs). The total fatty acid (TFA), SFA, MUFA, and PUFA contents of the Daya Bay population were significantly different from those of the Shanwei, Beihai, Nha Trang, and Dongshan populations (*p* < 0.05). Among the 13 fatty acids detected, palmitic acid (ranging from 0.494 to 1.068%) and linoleic acid (0.264 to 0.392%) had the highest contents, and the contents of these fatty acids were significantly greater in the Daya population than in the other populations (*p* < 0.05). The ratios of UFAs to TFAs ranged from 42.58 to 46.11%, with the highest ratios in the Beihai population and the lowest in the Shanwei population.

## 4. Discussion

### 4.1. Comparison of the C. lentillifera Basic Nutrient Compositions

The collection sites of the *C. lentillifera* samples in this study, which included the regions of Guangdong, Guangxi, Fujian, and Vietnam, span a wide range of geographical locations with environmental differences. In addition to moisture, the nutrient contents of the different populations of *C. lentillifera* were significantly different (*p* < 0.05). The ash content of the six populations of *C. lentillifera* ranged from 22.83 to 65.50%, which was in agreement with the results of Syakilla [22], and the ash contents in Guangxi, Fujian, and Vietnam were significantly greater than those in the three Guangdong populations, indicating that *C. lentillifera* could enrich the inorganic ions in the water [23]. Moreover, the crude protein, crude liquid, and total sugar contents of *C. lentillifera* ranged from 12.53 to 18.70%, 3.83 to 5.10%, and 18.13 to 32.87%, respectively, which was comparable to the ranges of 12.5 to 14.76%, 0.78 to 2.32%, and 21.32 to 50.71% reported in the study by Syakilla [22]. Furthermore, these values were essentially the same as those reported by Tang et al. [24] (9.22% crude protein and 0.81% crude liquid) and Wang et al. [25] (14.9% crude protein and 4.4% crude liquid). The crude protein, crude liquid, and total sugar contents of the Guangdong population were greater than those of the Guangxi, Fujian, and Vietnam populations, with the Daya Bay population being significantly different (*p* < 0.05) from the Beihai, Dongshan, and Nha Trang populations. The growth of *C. lentillifera* is affected by various factors, such as temperature, light, growth substrate, and growth cycle, and the differences in nutrient contents may be related to the cultivation environment, growth area, sampling time, and site [26]. Seaweeds, especially brown algae, are the main raw material sources from which mannitol and iodine are extracted in China [27]. In this study, the mannitol and iodine contents of *C. lentillifera* ranged from 5.30 to 7.47% and 0.03 to 0.07%, respectively, which was lower than those of kelp (12.3 to 15.1% and 0.36 to 0.47%, respectively) [28]. Thus, *C. lentillifera* is a suitable food to supplement the mannitol and iodine required by the human body but it is not suitable for use as a raw material for industrial extraction.

### 4.2. The Amino Acid Composition of C. lentillifera

Amino acids are the basic units of proteins, and the protein content and amino acid composition and content of *C. lentillifera* should be considered when evaluating the nutritional quality of this algae [29]. In this study, it was revealed that all six populations had the same amino acid composition, which is consistent with other studies on the nutrient composition of *C. lentillifera* [8]. When performing a nutritional evaluation, the FAO/WHO ideal protein model recommends that the ratio of EAAs to TAAs should be approximately 40% and that the EAA/NEAA ratio should be greater than 60% [29]. The amino acid compositions of five of the six regional populations, with the exception of the Daya population, conformed to the ideal protein model, suggesting that *C. lentillifera* is a high-quality protein source. Among the six regional populations in this study, the highest EAA/TAA and EAA/NEAA ratios were found in the Dongshan regional population, indicating that this population had highest quality protein, and the high contents of the DAAs Glu and Asp in the six populations were the source of the distinctive seaweed flavor of *C. lentillifera*. The highest DAA/TAA ratio was observed in the Beihai population, which indicated that this *C. lentillifera* population had the best fresh flavor. The DAA/TAA ratio was the highest in the Beihai population. Moreover, the proline (Pro) contents in the three Guangdong populations were significantly higher than those in the Beihai, Dongshan, and Nha Trang populations, and they differ from the amino acid contents reported in other studies. These previous studies on the nutrient composition of *C. lentillifera* revealed that proline is an osmotic regulator in plants and that environmental stresses cause an increase in the proline content in plants to improve plant resistance [30]. This study revealed that proline is an osmoregulatory substance in plants. The high proline content of *C. lentillifera* in Guangdong may be the result of an increase caused by environmental stress factors.

The level of satisfaction someone experiences from the food they consume is indicated by the taste parameter [31]. Enhancing the aroma and taste of food can be achieved by including glucose, sucrose, fiber, and other stimulants [32]. Non-volatile components in food are primarily responsible for the taste part of flavor, for example, lipids, polysaccharides, amino acids, and carbohydrates are responsible for the basic tastes of sweet, sour, salty, bitter, and umami in seaweeds [33]. Seaweeds are known for their ‘umami’ taste, which is considered the fifth basic taste, and this flavor is marked by organic acid, amino acid salts, and short peptides [34]. *C. lentillifera* from Daya Bay exhibited a higher protein content compared to populations from other regions, characterized by elevated levels of Asp (1.31 ± 0.19), Glu (1.18 ± 0.13), and gly (1.16 ± 0.19), which may enhance the palatability of the seaweed samples. This suggests the potential for developing *C. lentillifera* as a source of fresh flavorings.

### 4.3. A comprehensive Assessment of the Amino Acid Profile in C. lentillifera

The AAS results revealed that the first limiting amino acid of *C. lentillifera* was Met + Cys, and the second limiting amino acid was Ile or Lys, which was essentially consistent with the results of the study by Tang [24]. The EAAI is an indicator for evaluating the nutrient composition of food, and a high EAAI indicates high nutritional value. The EAAIs of the six populations in this study ranged from 45.89 to 61.09, among which the Nha Trang population had the highest EAAI, and thus, the highest nutritional value.

### 4.4. The Fatty Acid Composition of C. lentillifera

*C. lentillifera* is low in liquid but rich in fatty acids, with a total of 13 fatty acids detected, including six UFA species, with percentages ranging from 42.58% to 46.11%, which are higher than those reported by Wang [8], who reported values of 37.09% and 36.74%, respectively. Among the six populations in this study, the Daya Bay population presented the highest TFA and UFA contents, which were significantly greater than those of the Shanwei, Beihai, Dongshan, and Nha Trang populations (*p* < 0.05), whereas the Beihai population presented the highest percentage of UFAs. The fatty acid with the highest content was palmitic acid, which is consistent with the composition of seaweeds such as *Gracilaria lichenoides* and *Meristotheca papulosa* and the abundance of palmitic acid in nature [35]. In addition to palmitic acid, *C. lentillifera* is rich in PUFAs, with high contents of linoleic acid and Alpha linolenic acids, ranging from 0.155 to 0.329% and 0.133 to 0.296%, respectively. Linoleic acids and Alpha linolenic acids are essential fatty acids that cannot be produced by the human body but are important for preventing diseases and health maintenance [36]. In addition, the contents of arachidonic acids and EPA were high, at 0.038–0.105% and 0.017–0.055%, respectively. Arachidonic acid is essential for the development of the human brain and visual nerves and has important physiological activities. Terrestrial plants and animals rarely contain EPA, as it is mainly found in marine organisms, but EPA can reduce cholesterol and triglyceride contents and can promote the metabolism of saturated fatty acids in the body, thus reducing blood viscosity and preventing fat deposition in the blood vessel wall. It can also prevent cardiovascular diseases such as atherosclerosis, cerebral thrombosis, hypertension, and other cardiovascular diseases [37,38]. The World Health Organization’s guidelines on fatty acids recommend that both adults and children decrease their intake of SFA to constitute only 10% of their total energy consumption. Furthermore, it is advised to substitute saturated fatty acids with PUFA in the diet, as this substitution positively influences the regulation of blood cholesterol and triacylglycerol levels, thereby significantly reducing the risk of coronary heart disease [39,40]. According to the World Health Organization’s healthy dietary recommendations, the desiccated material of *C. lentillifera* exhibits potential for commercialization as a nutritious and appealing food product. For instance, snacks derived from the dried substance of *C. lentillifera* could be developed.

## 5. Conclusions

This study utilized samples collected from six farms located in Guangdong, Fujian, Guangxi, and Vietnam, and employed national standard methodologies for the quantification of nutrient compositions, including moisture, ash, crude protein, crude lipid, total sugar, mannitol, and iodine. The amino acid and fatty acid compositions of *C. lentillifera* from various cultivation regions were analyzed. The results of this study revealed that *C. lentillifera* is an edible seaweed rich in protein and total sugar but low in fat. It is a natural and healthy food because it is rich in amino acids present in composition ratios in accordance with FAO/WHO recommended standards. Additionally, *C. lentillifera* contains a relatively high percentage of a variety of UFAs, in addition to iodine and mannitol. This study demonstrated significant variation in the nutrient composition of *C. lentillifera* across different cultivation regions. Notably, *C. lentillifera* sourced from the Daya Bay exhibited superior nutritional value, suggesting enhanced health benefits. Additionally, *C. lentillifera* possesses a favorable flavor profile, rendering it suitable for consumption and its development into high-quality seafood condiment. This contributes to the environmentally sustainable advancement of *C. lentillifera* aquaculture.

## Figures and Tables

**Table 1:** Sample collection information.

Area	Sampling Point	Coordinates
Guangdong Province	Dapeng Bay of Penghu Sea area	114°28′48″ E, 22°31′12″ N
	Daya Bay of Huizhou	114°31′12″ E, 22°34′48″ N
	Great Lake of Shanwei	115°34′1″ E, 22°49′42″ N
Guangxi Province	North Sea	109°3′ E, 21°25′48″ N
Fujian Province	Dongshan	117°25′48″ E, 23°34′48″ N
Vietnam	Nha Trang	109°19′12″ E, 12°10′48″ N

**Table 2:** Nutritional components of *C. lentillifera* cultivated in different areas (*n* = 3, dry weight, %).

Cultivation Area	Guangdong Province			Guangxi Province	Fujian Province	Vietnam
	Dapeng Bay	Daya Bay	Shanwei	North Sea	Dongshan	Nha Trang
Moisture *	94.47 ± 0.51	94.83 ± 0.23	94.73 ± 0.81	94.87 ± 0.32	94.97 ± 0.35	94.57 ± 0.58
Ash	34.07 ± 1.34 ^b^	25.80 ± 0.70 ^bc^	22.83 ± 0.21 ^c^	57.90 ± 8.84 ^a^	59.60 ± 5.99 ^a^	65.50 ± 7.11 ^a^
Crude protein	18.70 ± 0.36 ^a^	18.57 ± 1.59 ^a^	17.20 ± 1.49 ^ab^	13.57 ± 3.33 ^bc^	14.17 ± 2.75 ^bc^	12.53 ± 1.16 ^c^
Crude liquid	4.57 ± 0.47 ^ab^	5.10 ± 0.36 ^a^	3.83 ± 0.81 ^b^	4.67 ± 0.25 ^ab^	3.93 ± 0.35 ^b^	4.27 ± 0.40 ^ab^
Total sugar	24.00 ± 3.38 ^b^	32.50 ± 4.22 ^a^	32.87 ± 2.59 ^a^	19.93 ± 1.93 ^bc^	20.00 ± 2.01 ^bc^	18.13 ± 2.50 ^c^
Mannitol	5.30 ± 1.15 ^b^	6.17 ± 1.46 ^ab^	7.17 ± 0.49 ^a^	6.57 ± 0.40 ^ab^	7.47 ± 0.25 ^a^	6.07 ± 0.25 ^ab^
Iodine	0.05 ± 0.02 ^ab^	0.03 ± 0.01 ^c^	0.04 ± 0.01 ^bc^	0.04 ± 0.00 ^bc^	0.05 ± 0.01 ^abc^	0.07 ± 0.00 ^a^

Notes: values with different letters indicate significant differences (*p* < 0.05); * indicates fresh weight.

**Table 3:** Amino acid composition of *C. lentillifera* cultivated in different areas (*n* = 3, dry weight, %).

Amino Acid	Guangdong Province			Guangxi Province	Fujian Province	Vietnam
	Dapeng Bay	Daya Bay	Shanwei	North Sea	Dongshan	Nha Trang
Thr *	0.77 ± 0.11 ^a^	0.82 ± 0.11 ^a^	0.81 ± 0.05 ^a^	0.44 ± 0.07 ^b^	0.40 ± 0.16 ^b^	0.49 ± 0.11 ^b^
Val *	0.57 ± 0.09 ^ab^	0.57 ± 0.11 ^ab^	0.68 ± 0.05 ^a^	0.49 ± 0.07 ^ab^	0.45 ± 0.15 ^b^	0.54 ± 0.12 ^ab^
Met	0.12 ± 0.06	0.15 ± 0.03	0.08 ± 0.04	0.12 ± 0.01	0.12 ± 0.04	0.12 ± 0.03
Ile *	0.47 ± 0.10	0.41 ± 0.08	0.51 ± 0.04	0.36 ± 0.06	0.35 ± 0.12	0.42 ± 0.10
Leu *	0.82 ± 0.20	0.78 ± 0.12	0.84 ± 0.06	0.62 ± 0.09	0.58 ± 0.20	0.70 ± 0.16
Phe *#	0.70 ± 0.12 ^ab^	0.71 ± 0.09 ^ab^	0.73 ± 0.04 ^a^	0.48 ± 0.08 ^c^	0.44 ± 0.16 ^c^	0.52 ± 0.12 ^bc^
Lys *	0.59 ± 0.07 ^ab^	0.66 ± 0.10 ^a^	0.63 ± 0.05 ^ab^	0.49 ± 0.08 ^ab^	0.43 ± 0.15 ^b^	0.53 ± 0.14 ^ab^
His *	0.30 ± 0.12 ^a^	0.31 ± 0.03 ^a^	0.35 ± 0.01 ^a^	0.15 ± 0.03 ^b^	0.12 ± 0.05 ^b^	0.16 ± 0.05 ^b^
Asp #	1.21 ± 0.15 ^ab^	1.31 ± 0.19 ^a^	1.32 ± 0.07 ^a^	0.86 ± 0.13 ^c^	0.80 ± 0.30 ^c^	0.96 ± 0.18 ^bc^
Ser	0.48 ± 0.07 ^ab^	0.55 ± 0.07 ^a^	0.51 ± 0.03 ^a^	0.30 ± 0.05 ^c^	0.29 ± 0.12 ^c^	0.34 ± 0.09 ^bc^
Glu #	1.10 ± 0.15 ^ab^	1.18 ± 0.13 ^ab^	1.21 ± 0.06 ^a^	0.94 ± 0.16 ^ab^	0.85 ± 0.28 ^b^	1.05 ± 0.20 ^ab^
Gly #	0.90 ± 0.19 ^abc^	1.16 ± 0.19 ^a^	1.07 ± 0.07 ^ab^	0.81 ± 0.09 ^bc^	0.67 ± 0.24 ^c^	0.79 ± 0.14 ^bc^
Ala #	0.29 ± 0.19 ^b^	0.59 ± 0.11 ^a^	0.60 ± 0.05 ^a^	0.49 ± 0.07 ^ab^	0.45 ± 0.15 ^ab^	0.56 ± 0.12 ^a^
Cys	0.26 ± 0.10 ^a^	0.27 ± 0.04 ^a^	0.23 ± 0.01 ^a^	0.09 ± 0.01 ^b^	0.08 ± 0.02 ^b^	0.08 ± 0.01 ^b^
Tyr #	0.37 ± 0.11	0.37 ± 0.05	0.30 ± 0.02	0.33 ± 0.05	0.31 ± 0.10	0.35 ± 0.09
Arg	0.46 ± 0.06 ^ab^	0.58 ± 0.06 ^a^	0.58 ± 0.03 ^a^	0.44 ± 0.06 ^ab^	0.38 ± 0.14 ^b^	0.46 ± 0.09 ^ab^
Pro	1.78 ± 0.21 ^a^	1.86 ± 0.19 ^a^	1.91 ± 0.14 ^a^	0.38 ± 0.06 ^b^	0.33 ± 0.11 ^b^	0.40 ± 0.09 ^b^
TAA	11.18 ± 1.68 ^ab^	12.27 ± 1.66 ^a^	12.37 ± 0.72 ^a^	7.77 ± 1.15 ^c^	7.05 ± 2.48 ^bc^	8.44 ± 1.75 ^c^
EAA	4.34 ± 0.84 ^ab^	4.41 ± 0.65 ^ab^	4.65 ± 0.32 ^a^	3.15 ± 0.49 ^bc^	2.89 ± 1.02 ^c^	3.47 ± 0.80 ^abc^
NEAA	6.84 ± 0.84 ^a^	7.87 ± 1.01 ^a^	7.73 ± 0.39 ^a^	4.63 ± 0.67 ^b^	4.15 ± 1.45 ^b^	4.99 ± 0.98 ^b^
DAA	4.57 ± 0.53 ^ab^	5.31 ± 0.75 ^a^	5.24 ± 0.25 ^a^	3.90 ± 0.57 ^ab^	3.52 ± 1.21 ^b^	4.23 ± 0.83 ^ab^
EAA/TAA	38.63 ± 1.63 ^b^	35.87 ± 0.51 ^c^	37.57 ± 0.45 ^b^	40.50 ± 0.27 ^a^	41.03 ± 0.21 ^a^	40.87 ± 0.91 ^a^
EAA/NEAA	63.00 ± 4.37 ^b^	55.93 ± 1.29 ^c^	60.10 ± 1.15 ^b^	68.10 ± 0.72 ^a^	69.63 ± 0.57 ^a^	69.23 ± 2.61 ^a^
DAA/TAA	41.00 ± 1.45 ^c^	43.23 ± 0.42 ^b^	42.30 ± 0.62 ^bc^	50.13 ± 0.15 ^a^	49.93 ± 0.29 ^a^	50.10 ± 0.82 ^a^

Notes: * Essential amino acids; # flavor amino acids. The values with different letters indicate significant differences (*p* < 0.05).

**Table 4:** Amino acid scores of *C. lentillifera* cultivated in different areas.

Evaluation Method	Amino Acid	Reference Proteins	Guangdong			Guangxi	Fujian Province	Vietnam
			Dapeng Bay	Daya Bay	Shanwei	North Sea	Dongshan	Nha Trang
AAS	Thr	250	257.35	276.03	295.53	202.70	177.93	242.70
	Val	310	190.51	192.99	248.29	225.74	197.07	269.28
	Met + Cys	220	127.24	142.49	115.19	96.74	86.03	99.24
	Ile	250	155.98	139.13	185.32	167.37	152.96	207.80
	Leu	440	272.96	262.57	306.43	287.15	257.34	347.42
	Phe + Tyr	380	431.15	458.95	496.58	446.87	385.28	523.61
	Lys	340	198.30	221.06	230.12	224.22	191.16	264.30
CS	Thr	292	88.13	94.53	101.21	69.42	60.93	83.12
	Val	411	46.35	46.96	60.41	54.92	47.95	65.52
	Met + Cys	386	32.96	36.92	29.84	25.06	22.29	25.71
	Ile	331	47.12	42.03	55.99	50.56	46.21	62.78
	Leu	534	51.12	49.17	57.38	53.77	48.19	65.06
	Phe + Tyr	565	76.31	81.23	87.89	79.09	68.19	92.67
	Lys	441	44.96	50.13	52.18	50.84	43.35	59.93
EAAI		100	52.59	54.23	59.65	52.12	45.89	61.09

**Table 5:** Comparative analysis of the fatty acid contents of *C. lentillifera* cultivated in different areas (*n* = 3, dry weight, %).

Fatty Acid	Guangdong Province			Guangxi Province	Fujian Province	Vietnam
	Dapeng Bay	Daya Bay	Shanwei	North Sea	Dongshan	Nha Trang
Myristic acids C14:0	0.022 ± 0.003 ^b^	0.042 ± 0.004 ^a^	0.036 ± 0.015 ^a^	0.006 ± 0.002 ^c^	0.009 ± 0.002 ^c^	0.009 ± 0.004 ^c^
Palmitic acids C16:0	0.886 ± 0.097 ^b^	1.068 ± 0.029 ^a^	0.853 ± 0.203 ^b^	0.661 ± 0.055 ^c^	0.537 ± 0.050 ^c^	0.494 ± 0.041 ^c^
Docosanoic acids C22:0	0.049 ± 0.005 ^a^	0.050 ± 0.007 ^a^	0.035 ± 0.006 ^b^	0.028 ± 0.002 ^bc^	0.020 ± 0.004 ^c^	0.019 ± 0.004 ^c^
Lignoceric acids C24:0	0.189 ± 0.019 ^a^	0.192 ± 0.023 ^a^	0.141 ± 0.026 ^b^	0.117 ± 0.011 ^b^	0.082 ± 0.013 ^c^	0.082 ± 0.009 ^c^
ΣSaturated fatty acids SFA	1.146 ± 0.123 ^ab^	1.325 ± 0.055 ^a^	1.065 ± 0.238 ^b^	0.813 ± 0.069 ^c^	0.648 ± 0.069 ^c^	0.604 ± 0.050 ^c^
Oleic acids (C18:1), n-9 *	0.069 ± 0.002 ^a^	0.068 ± 0.004 ^a^	0.071 ± 0.009 ^a^	0.033 ± 0.004 ^b^	0.025 ± 0.003 ^bc^	0.023 ± 0.005 ^c^
Erucic acids (C22:1), n-9 *	0.103 ± 0.014 ^a^	0.096 ± 0.034 ^a^	0.075 ± 0.010 ^ab^	0.032 ± 0.009 ^c^	0.021 ± 0.007 ^c^	0.027 ± 0.012 ^c^
ΣMonounsaturated fatty acids (MUFA)	0.079 ± 0.007 ^ab^	0.084 ± 0.002 ^a^	0.063 ± 0.020 ^bc^	0.054 ± 0.006 ^cd^	0.043 ± 0.005 ^d^	0.051 ± 0.003 ^cd^
Linoleic acids (C18:2), n-6 **	0.264 ± 0.024 ^b^	0.329 ± 0.011 ^a^	0.256 ± 0.067 ^b^	0.260 ± 0.037 ^b^	0.194 ± 0.017 ^c^	0.155 ± 0.014 ^c^
Cis−8,11,14−eicosatrienoic acids (C20:3), n-6 **	0.009 ± 0.001 ^b^	0.013 ± 0.000 ^a^	0.007 ± 0.002 ^c^	0.006 ± 0.001 ^c^	0.004 ± 0.001 ^d^	0.005 ± 0.000 ^d^
Arachidonic acids (C20:4), n-6 **	0.075 ± 0.005 ^b^	0.105 ± 0.002 ^a^	0.067 ± 0.017 ^b^	0.055 ± 0.007 ^bc^	0.040 ± 0.003 ^c^	0.038 ± 0.003 ^c^
Cis−11,14,17−eicosatrienoic acids (C20:3), n-3 **	0.007 ± 0.001 ^d^	0.008 ± 0.000 ^cd^	0.008 ± 0.001 ^cd^	0.020 ± 0.003 ^a^	0.012 ± 0.003 ^b^	0.011 ± 0.002 ^bc^
Eicosapentaenoic acids (C20:5; EPA), n-3 **	0.055 ± 0.004 ^a^	0.049 ± 0.001 ^a^	0.037 ± 0.012 ^b^	0.025 ± 0.003 ^c^	0.017 ± 0.001 ^c^	0.017 ± 0.001 ^c^
Alpha linolenic acids (C18:3), n-3 **	0.265 ± 0.029 ^ab^	0.296 ± 0.007 ^a^	0.206 ± 0.060 ^bc^	0.210 ± 0.036 ^bc^	0.153 ± 0.014 ^cd^	0.133 ± 0.017 ^d^
ΣPolyunsaturated fatty acids (PUFAs)	0.846 ± 0.057 ^ab^	0.964 ± 0.053 ^a^	0.727 ± 0.159 ^bc^	0.641 ± 0.089 ^cd^	0.466 ± 0.041 ^d^	0.407 ± 0.037 ^d^
TFA	2.072 ± 0.180 ^ab^	2.400 ± 0.097 ^a^	1.855 ± 0.413 ^bc^	1.507 ± 0.160 ^cd^	1.156 ± 0.115 ^de^	1.062 ± 0.089 ^e^
UFA/TFA	44.64%	43.66%	42.58%	46.11%	44.03%	43.13%

Notes: * monounsaturated fatty acids; ** polyunsaturated fatty acids; values with different letters indicate a significant difference (*p* < 0.05).

## Data Availability

The original contributions presented in the study are included in the article, further inquiries can be directed to the corresponding author.
